# Clinical characteristics of cerebral venous sinus thrombosis patients with new-onset of headache

**DOI:** 10.1186/s12883-023-03098-6

**Published:** 2023-02-02

**Authors:** Yugang Wang, Xiaozhu Shen, Ping Wang, Qi Fang

**Affiliations:** 1 Department of Neurology, The First Affiliated Hospital of Soochow University, Suzhou, Jiangsu China; 2 Department of Neurology, The First People’s Hospital of XianYang, XianYang, Sha’anxi China; 3 Department of Neurology, The Second People’s Hospital of Lian Yun Gang, Lian Yun Gang, Jiangsu, China; 4 Department of Neurology, The First People’s Hospital of He Fei, He Fei, An’Hui China

**Keywords:** Cerebral venous thrombosis, Clinical characteristics analysis, Headache, Clinical diagnosis

## Abstract

**Objective:**

This study aimed to assess the clinical characteristics of cerebral venous sinus thrombosis (CVT) patients with new-onset headache and to identify the risk factors for headache in this population.

**Methods:**

We retrospectively reviewed the demographic and clinical data of 69 CVT patients recruited between September 2017 and September 2019. Patients were classified into two groups, the headache group and the non-headache group, according to the presence or absence of new-onset headache symptoms at admission. The following characteristics and parameters were measured and analyzed, including gender, age, amount of thromboembolic cerebral venous sinus(ATCVS), and other relevant indicators.

**Results:**

The incidence of headache was 75% in this cohort. The proportion of female patients in the headache group was higher than that in the non-headache group. Patients in the headache group were younger than those without headache. CVT patients of headache group showed higher lymphocyte ratio (LR), blood urea nitrogen (BUN), and intracranial pressure (ICP) compared to the non-headache group, whereas mean corpuscular volume (MCV) and levels of protein (cerebrospinal fluid, CSF) and lactic dehydrogenase (LDH) in CSF were lower in headache patients. The data also revealed younger age and the increased level of chloride ion CI-(CSF) were the risk factors for the occurrence of headache in CVT patients.

**Conclusion:**

Age, LR, MCV, BUN levels, ICP, protein (CSF), and LDH (CSF) in patients with headache were significantly different from those in the non-headache group at admission. Younger age and a level of CI- (CSF) were risk factors for headache in CVT patients. These findings may provide guidance for clinical diagnosis and treatment of CVT.

## Introduction

Cerebral venous thrombosis (CVT) is an uncommon cerebrovascular disease that accounts for 0.5–2% of all stroke cases [1]. Patients with CVT often present with various symptoms including headache, dizziness, vomiting, nausea, seizures, unconsciousness, and unresponsiveness. CVT is also a rare, life-threatening disease that may cause sudden death [2]. It may occur in all age groups [3] and females are three times more likely to be diagnosed with CVT than males [4, 5]. The risk factors for CVT include infectious diseases (e.g. head and face infection) and non-infectious factors, such as hypercoagulability, blood stasis, head and neck trauma, oral contraceptive drugs, hormone replacement therapy, pregnancy [6] and low intracranial pressure [2]. No underlying risk factor is found in approximately 13% of CVT patients [7]. Current treatments for CVT include anticoagulation therapy, symptomatic therapy, and etiological treatment. Intravascular intervention may be applied to patients with severe CVT [8]. With the improvement in imaging diagnostic techniques and early treatment with anticoagulation, the mortality rate of CVT has decreased over the past years [9].

Headache is a common manifestation present in over 85% of CVT patients. However, there are limited studies that investigate the association between clinical characteristics of CVT patients and the occurrence of headache. The pathological mechanism of CVT-related headache may involve the stretching and compression of occluded venous sinuses, which lead to increased ICP and eventually pain in the head.

Different types of headaches have been observed in CVT patients, including exploding headache, migraine‑like headache, chronic tension headache, and chronic thunderclap headache [10, 11]. In this study, we analyzed the clinical characteristics of CVT patients with or without headache and identified the risk factors for headache in this population. Our findings may provide guidance for the early diagnosis of headache in patients with CVT.

## Methods

### Study design

A total of 69 consecutive patients who were diagnosed with CVT between September 2017 and September 2019 in the First Affiliated Hospital of Soochow University were recruited. The inclusion criteria were as follows: 1) patients who were diagnosed with CVT based on clinical presentation and imaging examinations including magnetic resonance venography (MRV), computed tomographic venography (CTV), or conventional digital subtraction angiography (DSA). In CTV, MRV, and DSA, CVT patients showed filling defects in cerebral venous sinuses. 2) patients were admitted to Neurology clinic. 3) patients were conscious, cooperative, and able to provide all necessary information. Patients were excluded if 1) they were diagnosed without imaging examinations; 2) they had arterial systematic cranial vascular disease, head trauma, acute intracranial infection, renal or hepatic failure, acute myocardial infarction, or hematological malignancy; 3) they were unwilling to cooperate or unable to provide reliable information.

**Fig. 1:**
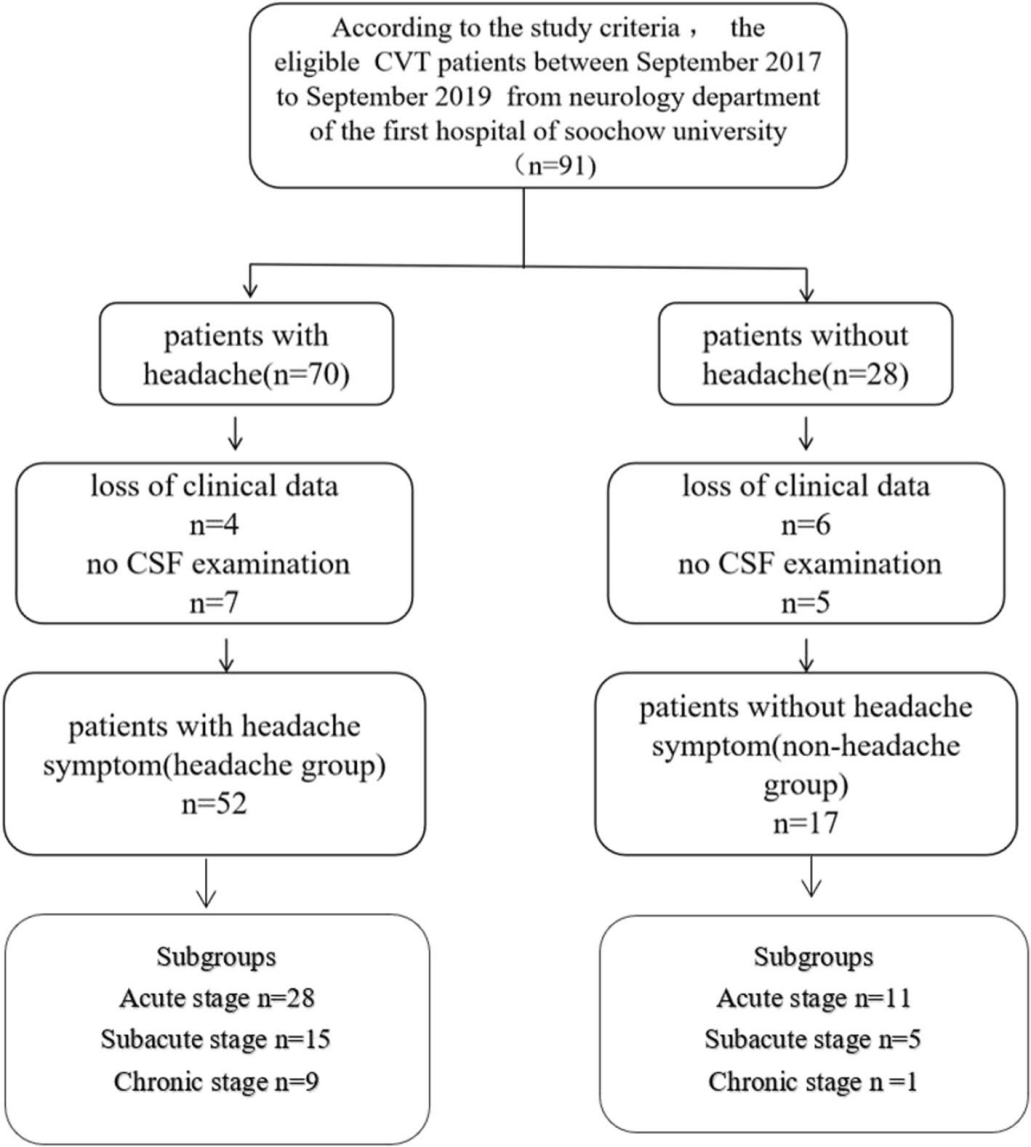
The flowchart of recruitment and selection process, there are total 91patients information collected in our study, but 22 patients were excluded for loss clinical data and other reasons. Sixty-nine patients were into the research lastly

The patients were divided into two groups, the headache group and the non-headache group, according to the presence or absence of new-onset headache symptoms at admission. Headache was defined as a pain on the top of the head, in the forehead, or an occipital ache. Patients in each group were further classified into three subgroups based on the stage of CVT: 1) acute stage: ≤ 48 h; 2) subacute stage: between 48 h and 30 days; 3) chronic stage, ≥ 30 days [12]. All participants received formal anticoagulation therapy. Patients with severer CVT also received intravascular treatment. This study was approved by the ethics committee of the hospital. All patients provided written informed consent (Fig. 1).

### Data collection

The demographic and clinical data of 69 CVT patients were collected and retrospectively reviewed. The characteristics of patients with or without headache were compared, including demographic features, date of symptom onset, all symptoms presented from the onset of the headache at admission, the results from imaging examinations, and the National Institutes of Health Stroke Score (NIHSS) at admission and at discharge, Patient outcomes were assessed at discharge by NIHSS. The following parameters, which were measured within 24 h after admission.The patients systolic blood pressure(SBP), diastolic blood pressure(DBP)were measuered and analyzed in 24 h after admission.Amount of thromboembolic cerebral venous sinus were counted after have a MRV check. levels of D-dimer and hypersensitive C-reactive protein, white blood cell count, the number of lymphocytes(L), lymphocytes ratio(LR), Mean corpuscular volume (MCV), levels of hepatitis B surface antibody and blood urea nitrogen(BUN) in peripheral blood in 24 h after admission. the pressure of the cerebrospinal fluid (CSF), levels of protein, Lactic dehydrogenase(LDH), and adenosine deaminase(ADA)in the CSF were anayzed after undergoing a lumbar puncture in 48 h after admission. urine specific gravity (USG) was detected in 24 h after admission, Patient outcomes were assessed by two professional Physician of neurology at discharge by NIHSS. The severity of CVT was determined by amount of thromboembolic cerebral venous sinus. The severity of headache was evaluated using Visual Analogue Scale-100(VAS-100). An ICP of more than 200 mmH_2_O was defined as intracranial hypertension.

### Statistical analysis

All data analyses were performed by SPSS (version 21, IBM). Quantitative variables that were normally distributed were expressed as mean ± standard deviation, whereas non-normally distributed data were shown as median with inter-quartile range (IQR). Categorical variables were presented as number and percentage (%). Student’s *t*-test or Mann–Whitney test was used for the analysis of continuous data, while χ^2^ or Fisher’s exact test was used to compare categorical data. The independent variables associated with headache and the risk factors for CVT were analyzed by logistic regression analysis. A difference was considered significant if *P* < 0.05.

## Results

The overall incidence of headache was 75% (52/69) in this cohort. In the headache group (*n* = 52), 55.8% (29/52) of the patients were females. The median age was 37 years [IQR 27–45 years]. Among these patients, 28 (51.9%) had exploding headache; 15 (28.8%) had migraine‑like headache; 3 (5.7%) had chronic tension headache 3; 6 (11.54%) had other types of headache. In the non-headache group (*n* = 17), 5 (29%) of the patients were females. The median age of patients without headache was 50 years [IQR 38–67 years] (Table 1).

**Table 1:** Demographic and clinical characteristics of patients at admission

	Headache groups *n* = 52	Non-headache groups *n* = 17	*X*^*2*^*/Z*-value	*P*-value
Gender (male/female)	23/29	12/5	4.947	0.025
Age (years)	37 (27, 45)	50 (38, 67)	-2.862	0.004
Subgroups				
Acute stage	28 (53.84%)	11 (64.70%)	1.423	0.0491
Subacute stage	15 (28.85%)	5 (29.41%)		
Chronic stage	9 (17.31%)	1 (9.09%)		
ATCVS	3 (1, 4)	3 (1, 3)	-0.732	0.670
SBP (mmHg)	121 (112, 131)	129 (120, 141)	-1.928	0.054
DBP (mmHg)	74 (67, 80)	78.5 (69, 89.75)	-1.132	0.258
WBC (× 10^9^/L)	7.33 (5.78, 8.92)	8.05 (6.49, 11.06)	-1.436	0.151
L (× 10^9^/L)	1.7 (1.09, 2.08)	1.39 (1.05, 2.24)	-0.555	0.579
LR (%)	0.25(0.172,0.327)	0.18 (0.14, 0.236)	-2.076	0.038
MCV (ng/L)	88.10 (84, 91)	90.4 (88.65, 92.40)	-2.112	0.035
USG	1.014 (1.010, 1.020)	1.013 (1.010, 1.015)	-0.993	0.479
HBs-Ab (ug/ml)	24.03(24.03,154.54)	28.07 (5.97,154.56)	-0.271	0.787
BUN (mg/L)	3.5 (2.80, 4.15)	4.75 (3.90, 6.00)	-3.052	0.002
ICP (mm H_2_O)	234 (200, 290)	200 (171.25, 234)	-2.237	0.025
Protein (CSF) (g/L)	0.55 (0.284, 0.605)	0.605 (0.605, 0.995)	-3.103	0.002
ADA (CSF) (U/L)	0.4 (0.20, 0.73)	0.73 (0.40, 0.73)	-1.638	0.101
LDH (CSF) (U/L)	28 (22, 54)	50 (34, 54)	-2.004	0.045
CL (CSF) (mmonl/L)	121.50 (120.70, 125.30)	120.70 (120.70, 123.67)	-1.384	0.166
NHISS (at discharge)	0.00 (0, 1)	0.5 (0, 1.75)	-1.798	0.072
Treatment method				
Low molecular heparin	50 (96.15%)	17 (100%)	0.673	0.412
Intravascular treatment	2 (3.84%)	0 (0)		
Prognosis				
Improved	50 (96.15%)	14 (82.35%)	9.393	0.024
Exacerbation/complications	1 (1.92%)	3 (17.65%)		
Death	1 (1.92%)	0 (0)		

*ATCVS* Amount of thromboembolic cerebral venous sinus, *SBP* Systolic blood pressure, *DBP* Diastolic blood pressure, *WBC* White blood cell count (× 10^9^/L), *LR* Lymphocyte ratio, *MCV* Mean corpuscular volume, *USG* Urine specific gravity, *HBs-Ab* Hepatitis B surface antibody, *BUN* Blood urea nitrogen, *ICP* Intracranial pressure, *CSF* Cerebrospinal fluid, *ADA* Adenosine deaminase, *LDH* Lactic dehydrogenase, *NHISS* the National Institutes of Health Stroke Scale, *LWMH* Low molecular heparin

Among all CVT patients, 47.82% of them were at the acute stage; 33.33% were at the subacute stage; and 18.84% were at the chronic stage. The proportions of headache patients at acute, subacute, and chronic stages were 53.84% (28/52), 28.84% (15/52), and 17.30% (15/52), respectively. In the non-headache group, the percentage of patients at acute, subacute, and chronic stages were 64.70% (11/17), 29.41% (5/11), 9%(1/11), respectively.

Head and face infection or upper respiratory infection occurred in 27.68% (26/69) of all CVT patients. There was no significant difference in the NHISS between headache and non-headache groups (0.0 [0.0, 1.00] vs. 0.5 [0.0, 1.75], *P* = 0.072). Among all participants, 67 of them received anticoagulant therapy (low molecular heparin, 5000 IU, subcutaneous injection, b.i.d.) for two weeks. Two patients received intravascular treatment. At discharge, 92.75% of all patients had an NIHSS of 0, indicating that they were in relatively good condition; 7.25% of them were in a relatively poor condition (NHISS > 1); 4 patients were in coma. One patient from the head group died during hospitalization (Table 1). The result of VAS-100 in patients with headache was 70.12 ± 13.8.

**Fig. 2:**
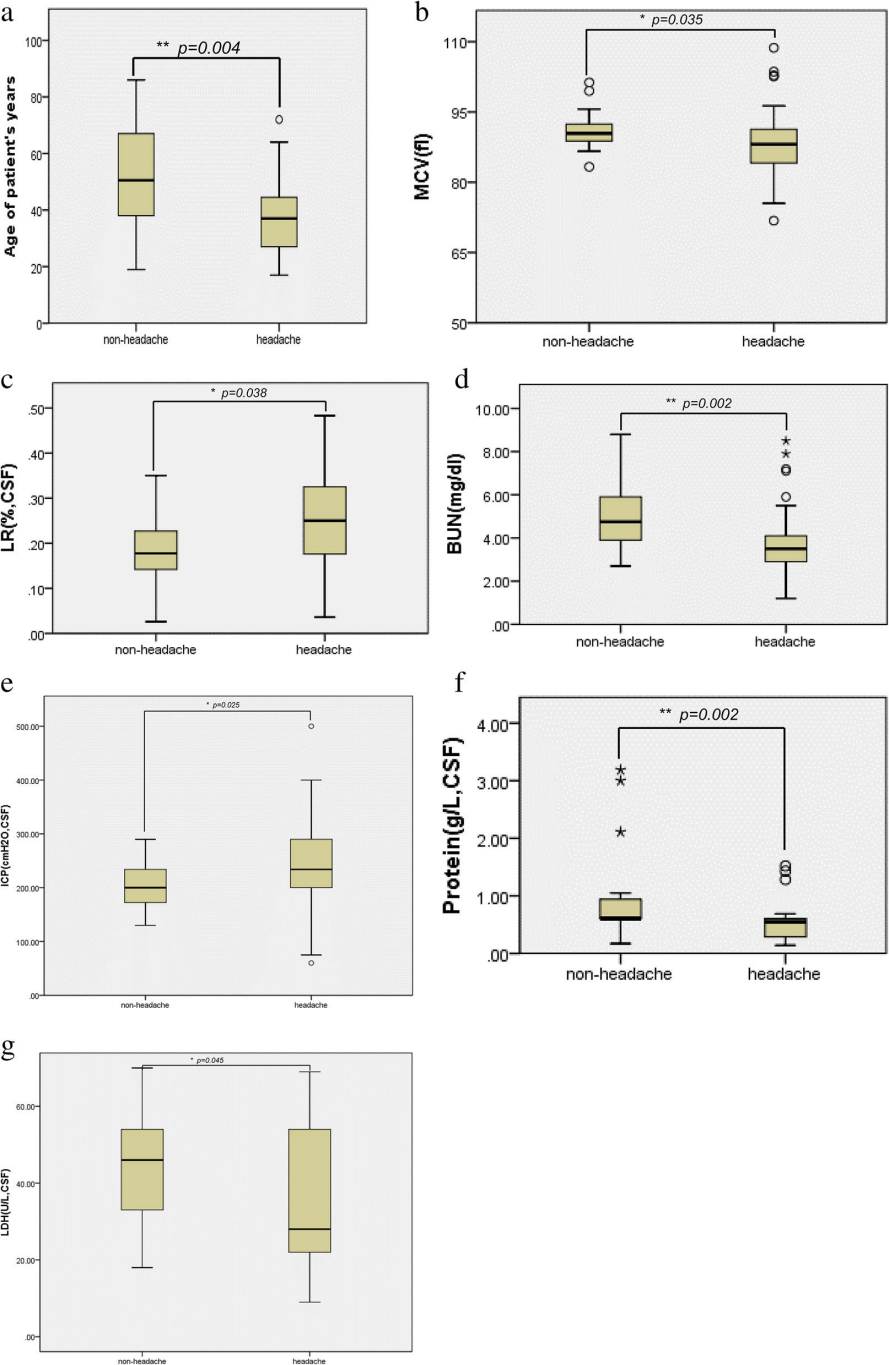
The comparsion of clinical characteristics of patients at admission between two groups (head-group & non-headgroup). ***a***, the age (*p* = *0.004,*), ***b***, the MCV (*p = *0.035*), ***c***, LR (*p = *0.038*),*** d***, the BUN ( *p = *0.002*), ***e***, the ICP (*p = *0.025*), ***f***,protein(CSF) (*p = *0.002*), ***g****,*LDH(CSF) (*p = *0.045*). LR: Lymphocyte ratio, MCV: Mean corpuscular volume, BUN: Blood urea nitrogen, ICP: Intracranial pressure, CSF: Cerebrospinal fluid, LDH: Lactic dehydrogenase. (*p* ≤ *0.05means significantly difference)*

Compared with the non-headache group, patients with headache had lower median age (37 [27, 45] vs.50 [38, 67], *P* = 0.004), MCV (88.10 [84, 91] vs. 90.4 [88.65, 92.40], *P* = 0.035), levels of BUN (3.5 [2.80, 4.15] vs. 4.75 [3.90, 6.00], *P* = 0.002), protein (CSF) (0.55 [0.284, 0.605] vs. 0.605 [0.605, 0.995], *P* = 0.002), LDH (CSF) (28 [22, 54] vs. 50 [34, 54], *P* = 0.045), and higher LR (0.25 [0.17, 0.32] vs. 0.17 [0.14, 0.24], *P* = 0.038) and ICP (234 [200, 290] vs. 200 [171.25, 234], *P* = 0.025). However, there was no significant difference in NHISS, ATCVS, DBP, WBC, L, USG, level of ADA (CSF), and treatment method between the two groups. The headache group had better outcomes at discharge (*X*^*2*^ = 9.393, *P* = 0.024) (Table 1, Fig. 2).

The Spearman’s correlation analysis showed that the younger age (*r* = 0.352, *P* = 0.003), gender of female (*r* = 0.272, *P* = 0.026), increased LR (*r* = 0.252, *P* = 0.037) and ICP (*r* = 0.271,* p* = 0.024) were positively correlated with the occurrence of headache, whereas decreased MCV (*r* = -0.256, *P* = 0.034), lower levels of BUN (*r* = -0.370, *P* = 0.002) and protein (CSF) (*r* = -0.376, *P* = 0.001) were negatively correlated with the onset of headache in CVT patients (Table 2).

**Table 2:** Correlations between demographic/clinical characteristics and the occurrence of headache in CVT patients

	*r-value*	*P-value*
Age (years)	0.352	0.003
Gender	0.272	0.026
LR(%)	0.252	0.037
ICP (mg/L)	0.271	0.024
BUN (mg/L)	-0.370	0.002
MCV(ng/L)	- 0.256	0.034
Protein (CSF)(g/L)	- 0.376	0.001

*LR* Lymphocyte ratio, *MCV* Mean corpuscular Volume, *BUN* Blood urea nitrogen, *ICP* Intracranial pressure, *CSF* Cerebrospinal fluid, *r* Relation coefficient

We further performed binary logistic regression analysis to identify the risk factors for headache in CVT patients. The occurrence of headache was defined as a dependent variable and the independent variables included DBP, SBP, NHISS, WBC, L, LR, MCV, USG, ICP, levels of HBs-Ab, BUN, protein (CSF), ADA (CSF), LDH (CSF), CI- (CSF), and NHISS (at discharge). The results showed that younger age (adjusted odds ratio (OR) = 0.912, 95% confidence interval (*CI*): 0.840–0.990, *P* = 0.029] and the increased level of CI- (CSF) (adjusted OR = 1.742, 95% *CI*: 1.037–2.927, *P* = 0.036) were the predictive factors for the occurrence of headache in CVT patients (Table 3).

**Table 3:** Binary logistic regression analysis

	*B*	*S.E*	*Walds*	*p-value*	*Exp (B)*	*EXP(B) 95% C.I*	
Gender	1.775	1.756	1.022	0.312	5.902	0.189	184.455
Age(years)	-.0092	0.042	4.789	0.029	0.912	0.840	0.990
SBP (mmHg)	-0.104	0.056	3.442	0.064	0.901	0.807	1.006
DBP (mmHg)	0.078	0.055	2.063	0.151	1.082	0.972	1.204
NHISS	-0.155	0.219	0.499	0.480	0.857	0.557	1.316
WBC(× 10^9^/L)	0.282	0.312	0.819	0.365	1.326	0.720	2.443
PLT(× 10^12^/L)	-0.010	0.007	2.132	0.144	0.990	0.978	1.003
ATCVS	0.046	0.465	.010	0.922	1.047	0.421	2.605
ICP(mg/L)	0.015	0.011	2.130	0.144	1.016	0.995	1.037
CL^−^(CSF) (mmonl/L)	0.555	0.265	4.393	0.036	1.742	1.037	2.927
LDH(CSF) (U/L)	-0.006	0.011	.258	0.612	.994	0.973	1.016
BUN(mg/L)	-0.101	0.538	0.035	0.851	0.904	0.315	2.594
L(× 10^9^/L)	-1.929	1.779	1.176	0.278	0.145	0.004	4.747
PT(second)	0.163	0.303	0.289	0.591	1.177	0.650	2.133
Fibrinogen(g/L)	-0.130	0.428	0.093	0.761	0.878	0.380	2.030

*SBP* Systolic blood pressure, *DBP* Diastolic blood pressure, *NHISS* the National Institutes of Health Stroke Scale, *WBC (*× *10*^*9*^*/L)* White blood cell count, *PLT* Blood platelet, *ATCVS* Amount of thromboembolic cerebral venous sinus, *ICP* Intracranial pressure, *CSF* Cerebrospinal fluid, *LDH* Lactic dehydrogenase *BUN* Blood urea nitrogen, *L* Lymphocyte, *PT* Prothrombin time

**Fig. 3:**
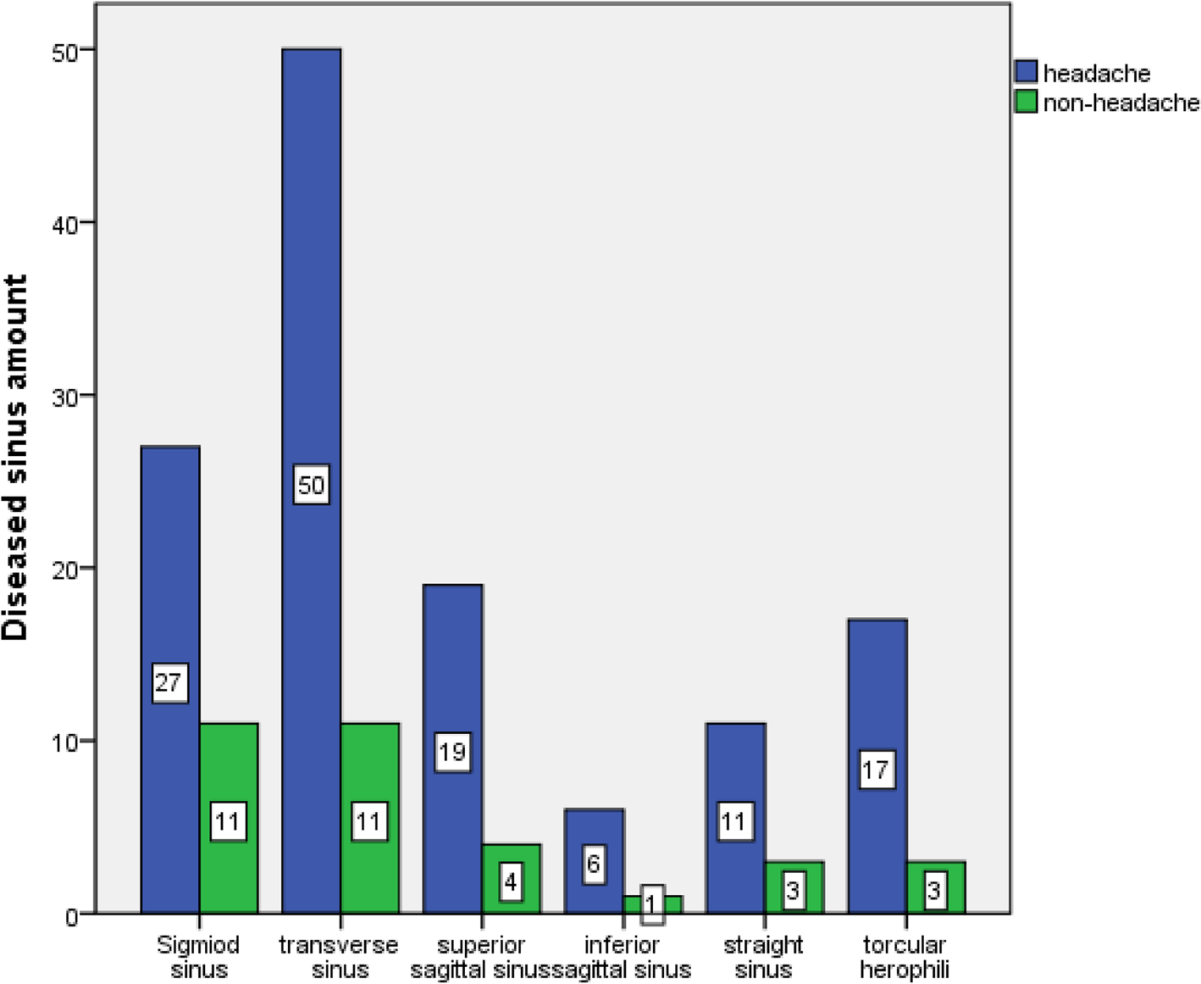
Differences of ATCVS, amount of thromboembolic cerebral venous sinus (diseased sinus) between two groups (head-group & non-headgroup) were significant. The figure shown sigmoid sinus 27(20.7%), transverse sinus 50(38.4%), superior sagittal sinus19(14.6%), inferior sagittal sinus 6(4.6%), torcular herophili 11(8.4%), straight sinus 17 (13.1%) in the headache group patients, and sigmoid sinus 11(33.4%), transverse sinus11(33.4%), superior sagittal sinus 4(12.1%), inferior sagittal sinus 1(3.0%), torcular herophili 3 (9.0%), straight sinus 3(9.0%) in the non-headache group patients (*p* ≤ *0.05 means significantly difference)*

We found the amount(percentage) of thromboembolic cerebral venous sinus were sigmoid sinus 27(20.7%), transverse sinus 50(38.4%), superior sagittal sinus 19(14.6%), inferior sagittal sinus 6(4.6%), torcular herophili 11(8.4%), straight sinus 17 (13.1%) in the headache group patients, and sigmoid sinus 11(33.4%), transverse sinus11(33.4%), superior sagittal sinus 4(12.1%), inferior sagittal sinus 1(3.0%), torcular herophili 3 (9.0%), straight sinus 3(9.0%) in the non-headache group patients (Fig. 3).

## Discussion

Headache is one of the most common symptoms in CVT patients. In this cohort, 75% (52/69) of the patients reported headache at admission, which was consistent with a previous study showing that 80–90% of CVT patients presented with headache [13]. Previous evidence reveals that CVT is most prevalent among young women [14]. Here, we found that patients with headache were younger than those in the non-headache group. Also, 78.7% of our patients were females. It has been reported that headache is associated with papilledema in 25–75% of CVT patients [15]. The proportions of female patients in headache and non-headache groups were 55.67% and 29.42%, respectively. Headache may occur at the acute, subacute, or chronic stages of CVT. Botta et al*.* found that headache onset was acute in 51.1%, subacute in 42.6%, thunderclap in 4.3%, and chronic in 2.1% of the CVT patients and the mean VAS was 76.4 ± 18.8 [10], which was consistent with our findings.

Infection has been considered as one of the most common causes of CVT [16] and the neutrophil-to-lymphocyte ratio is significantly associated with poor outcomes at discharge [16, 17]. Our study showed that head and face infection or upper respiratory infection occurred in 27.68% (26/69) of the patients. Compared to the non-headache group, patients with headache had a higher LR. However, no significant difference was observed in other inflammation markers, such as WBC and the number of lymphocytes. We also found that a high level of HBs-Ab in all CVT patients, but there was no significant difference between the headache and non-headache groups. Previous studies reported that CVT patients had a higher HBs-Ag-positive rate compared to health controls [16, 18], indicating HBV infection may be a risk factor for CVT [18].

Intracranial CSF pressure is an important sign of CVT [1, 19]. In the current study, the ICP exceeded 200 mm H_2_O in both groups. Also, CVT patients with headache showed higher ICP compared to the non-headache group. Intracranial hypertension caused indirectly by a mass compressing part of the intracranial venous sinuses, resulting in obstruction of venous drainage [17].

CSF examination provides important information for the diagnosis of CVT. Most CVT patients have normal or increased cell counts and protein concentrations in the CSF [1]. In our cohort, the headache group showed significantly lower levels of protein (CSF), Cerebrospinal fluid protein content is increased in all patients with cerebral venous sinus thrombosis. Compared with non-headache patients, cerebrospinal fluid protein content is relatively lower in headache patients, which may be related to increased protein catabolism or poor nutrition in headache patients. ADA (CSF), LDH (CSF), and CI- (CSF) also were lower compared to patients non-headache. The changes in CSF profile in headache patients may be related to the inflammation of sensory nerves. We also demonstrated that the MCV in the headache group was significantly lower than that in patients without headache. Anemia caused by iron deficiency is a common disorder in juvenile populations [20]. Iron-deficiency anemia has been identified as a contributor to the development of CVT [21]. Some patients have normal hemoglobin, and although the MCV is lower, we have found that red blood cells are small and prone to suffer CVT. Hypercoagulability and venous stasis have also been shown to play vital roles in the pathogenesis of thrombus [4]. Further investigations are needed to explore the relationships between these clinical parameters and headache in CVT patients.

There are some limitations in this study. First, due to the low incidence of CVT. The number of recruited patients was relatively small, which may not present the whole of CVT patients in China. Also, we did not divide headache patients based on types, severity, or symptoms of the pain. Multicenter studies with a larger sample size and more subgroups will be performed in the future.

## Conclusion

The age, LR, MCV, levels of BUN, ICP, protein (CSF), and LDH (CSF) in headache patients were significantly different from those in the non-headache group. Younger age and a lower level of CI- (CSF) were risk factors for the occurrence of headache in CVT patients. These findings may provide guidance for clinical diagnosis and treatment of CVT.

## Data Availability

The datasets generated and/or analysed during the current study are not publicly available due to containing private information such as age and gender but are available from the corresponding author on reasonable request.

## Abbreviations

CVT: Cerebral venous sinus thrombosis
ATCVS: Amount of thromboembolic cerebral venous sinus
SBP: Systolic blood pressure
DBP: Diastolic blood pressure
WBC: White blood cell count (× 10^9^/L)
LR: Lymphocyte ratio
MCV: Mean corpuscular volume
USG: Urine specific gravity
HBs-Ab: Hepatitis B surface antibody
BUN: Blood urea nitrogen
ICP: Intracranial pressure
CSF: Cerebrospinal fluid
ADA: Adenosine deaminase
LDH: Lactic dehydrogenase
NHISS: The National Institutes of Health Stroke Scale
LWMH: Low molecular heparin

## References

[1] Wang X, Sun X, Liu H (2012). Clinical analysis and misdiagnosis of cerebral venous thrombosis. Exp Ther Med.

[2] Fan Y, Yu J, Chen H, Zhang J, Duan J, Mo D, Zhu W, Wang B, Ouyang F, Chen Y (2020). Chinese Stroke Association guidelines for clinical management of cerebrovascular disorders: executive summary and 2019 update of clinical management of cerebral venous sinus thrombosis. Stroke Vasc Neurol..

[3] Zuurbier SM, Hiltunen S, Lindgren E, Silvis SM, Jood K, Devasagayam S, Kleinig TJ, Silver FL, Mandell DM, Putaala J (2018). Cerebral venous thrombosis in older patients. Stroke.

[4] Bibi A, Liyanapthirana C, Khan S: Rare presentation of iron deficiency anaemia with cerebral venous sinus thrombosis in a middle-aged woman. BMJ Case Rep. 2019;12(1):2. 10.1136/bcr-2018-225851 PMC6340589 30659005

[5] Coutinho JM, Ferro JM, Canhao P, et al. Cerebral venous and sinus thrombosis in women[J]. Stroke. 2009;40(7):2356–61. 10.1161/STROKEAHA.108.543884. 10.1161/STROKEAHA.108.543884 19478226

[6] Lo YC, Tsai JL, Tsai IT, Hsu CW (2016). Headache, oestrogens, homocysteinaemia and cerebral venous thrombosis. QJM.

[7] José M. Ferro M, PhD; PatríciaCanhão, MD; Jan Stam, MD;, Marie-Germaine Bousser MFB, MD;: Prognosis of Cerebral Vein and Dural Sinus ThrombosisResults of the International Study on Cerebral Vein and Dural SinusThrombosis (ISCVT). Stroke. 2003. 10.1161/01.STR.0000117571.76197.26. 10.1161/01.STR.0000117571.76197.26 14976332

[8] Bushnaq SA, Qeadan F, Thacker T, Abbas M, Carlson AP (2018). High-risk features of delayed clinical progression in cerebral venous thrombosis: a proposed prediction score for early intervention. Interv Neurol.

[9] Unal AY, Unal A, Goksu E, Arslan S (2016). Cerebral venous thrombosis presenting with headache only and misdiagnosed as subarachnoid hemorrhage. World J Emerg Med.

[10] Botta R, Donirpathi S, Yadav R, Kulkarni GB, Kumar MV, Nagaraja D (2017). Headache patterns in cerebral venous sinus thrombosis. J Neurosci Rural Pract.

[11] de Bruijn S, Stam J, Kappelle LJ (1996). Thunderclap headache as first symptom of cerebral venous sinus thrombosis. The Lancet.

[12] Idiculla PS, Gurala D, Palanisamy M, Vijayakumar R, Dhandapani S, Nagarajan E (2020). Cerebral venous thrombosis: a comprehensive review. Eur Neurol.

[13] Singh RJ, Saini J, Varadharajan S, Kulkarni GB, Veerendrakumar M (2018). Headache in cerebral venous sinus thrombosis revisited: exploring the role of vascular congestion and cortical vein thrombosis. Cephalalgia.

[14] Gunes HN, Cokal BG, Guler SK, Yoldas TK, Malkan UY, Demircan CS, Yon MI, Yoldas Z, Gunes G, Haznedaroglu IC (2016). Clinical associations, biological risk factors and outcomes of cerebral venous sinus thrombosis. J Int Med Res.

[15] Kulkarni GB, Singh RJ, Gadad V, Ramakrishnan S, Mustare V (2019). Unilateral papilledema in cerebral venous sinus thrombosis. J Neurosci Rural Pract.

[16] Wang L, Duan J, Bian T, Meng R, Wu L, Zhang Z, Zhang X, Wang C, Ji X (2018). Inflammation is correlated with severity and outcome of cerebral venous thrombosis. J Neuroinflammation.

[17] Park JH, Yoon SH (2008). New concept of cerebrospinal fluid dynamics in cerebral venous sinus thrombosis. Med Hypotheses.

[18] Shan F, Gao C, Long Y, Huang L, Zheng Y, Chen M, Fan Y, Yin J (2015). Cerebral venous sinus thrombosis may be associated with hepatitis B virus infection: a preliminary finding. Neurol Res.

[19] Zhang D, Wang J, Zhang Q, He F, Hu X (2018). Cerebral Venous Thrombosis in Spontaneous Intracranial Hypotension: A Report on 4 Cases and a Review of the Literature. Headache.

[20] Machen L, Abbasian J (2019). Cerebral venous sinus thrombosis and iron deficiency anemia presenting as bilateral disc edema in a child. Can J Ophthalmol.

[21] Kamel WA, Al-Hashel JY, Alexander KJ, Massoud F, Al Shawaf F, Al Huwaidi IE (2017). Cerebral venous thrombosis in a patient with iron deficiency anemia and thrombocytopenia: a case report. Open Access Macedonian J Med Sci.

